# Comparative biodegradation of functionalized graphene oxide nanosheets by myeloperoxidase and neutrophil extracellular traps

**DOI:** 10.3389/fbioe.2026.1797692

**Published:** 2026-05-13

**Authors:** Mohsen Bahmani, Asma Gholami, Marziyeh Ghaeidamini, Zhejian Cao, Mohammad Reza Gharib-Zahedi, Pallab Sanpui, Elin K. Esbjörner, Leif A. Eriksson, Ivan Mijakovic, Shadi Rahimi

**Affiliations:** 1 Division of Systems and Synthetic Biology, Department of Life Sciences, Chalmers University of Technology, Gothenburg, Sweden; 2 Sapienza University of Rome, Rome, Italy; 3 Department of Chemistry and Molecular Biology, University of Gothenburg, Gothenburg, Sweden; 4 Division of Molecular Bioscience, Department of Life Sciences, Chalmers University of Technology, Gothenburg, Sweden; 5 Department of Molecular Sciences, Swedish University of Agricultural Sciences (SLU), Uppsala, Sweden; 6 Department of Biotechnology, Birla Institute of Technology and Science, Dubai, United Arab Emirates; 7 The Novo Nordisk Foundation Biotechnology Research Institute for the Green Transition, Technical University of Denmark, Lyngby, Denmark

**Keywords:** biodegradation, graphene oxide, molecular dynamics simulations, neutrophil extracellular traps, neutrophils

## Abstract

**Introduction:**

Graphene oxide (GO) nanosheets have attracted significant interest as potential carriers for drug delivery due to their unique physicochemical properties and large surface area. However, concerns regarding their cytotoxicity and biodegradability must be addressed before clinical translation. In this study, we aimed to evaluate the biocompatibility and biodegradation of GO functionalized with polyethylene glycol (PEG) and polyethyleneimine (PEI), two commonly used polymers in biomedical applications.

**Method:**

The interactions of GO, GO-PEG, and GO-PEI with granulocyte-like cells were investigated to determine their effects on cell viability and their susceptibility to immune-mediated degradation. Biodegradation of the materials was assessed using Raman spectroscopy after exposure to granulocyte-like cells, neutrophil extracellular traps (NETs), and myeloperoxidase (MPO), a key enzyme present in NETs. In addition, circular dichroism (CD) spectroscopy was used to evaluate structural changes in MPO upon interaction with the materials, and molecular dynamics simulations were performed to investigate the interaction of hypochlorous acid (HOCl), the catalytic product of MPO, with GO and its functionalized derivatives.

**Results and discussion:**

The results showed that functionalization with PEG or PEI significantly improved cell viability compared with pristine GO. Although GO was structurally modified by granulocyte-like cells, NETs, and MPO, GO-PEG did not show significant degradation under these conditions. In contrast, GO-PEI was susceptible to structural modification by both NETs and MPO. CD analysis indicated that MPO maintained a more stable secondary structure in the presence of GO-PEI compared with GO or GO-PEG under oxidative conditions, suggesting that MPO-generated HOCl may play a key role in GO-PEI degradation. Molecular dynamics simulations further demonstrated stronger interaction and retention of HOCl in GO-PEG and GO-PEI systems compared with pristine GO, indicating an enhanced interaction between oxidants and the functionalized materials. Overall, these findings demonstrate that polymer functionalization significantly influences the biocompatibility and immune-mediated structural modification of GO. Importantly, this study provides new mechanistic insights into how PEG and PEI modifications affect MPO-driven oxidative degradation pathways of graphene oxide-based nanomaterials. These results highlight GO-PEI as a biodegradable and biocompatible candidate for future biomedical and drug delivery applications.

## 1 Introduction

The unique physical and chemical properties of graphene and its derivatives (graphene-based materials) have led to diverse applications of these materials. The most prominent biomedical applications of graphene-based materials are in drug delivery and prevention of biofilm formation (Chen et al., 2021; Rahimi et al., 2022; Pandit et al., 2021; Rahimi et al., 2023a; Rahimi et al., 2023b). The oxygenated derivative of graphene, graphene oxide (GO), has particularly interesting features for drug delivery (Rahimi et al., 2023b). It contains alcohol, carboxyl, and epoxide functional groups, which result in higher water dispersibility of GO and can be used as chemical handles for covalent modifications. Moreover, it has been proposed that the water layer associated to oxidation sites of hydrophilic functional groups on the GO surface enhances the biocompatibility of this material and even stimulates the growth and proliferation of mammalian cells in contact with GO (Ruiz et al., 2011; Zhao et al., 2016). Despite these promising properties, the clinical translation of graphene-based materials requires careful evaluation of their biocompatibility and potential toxicity (Lanai et al., 2024).

Previous studies have shown that pristine graphene (pG)-based nanomaterials may exhibit size-, dose-, and time-dependent toxicity toward various cell types. Such toxicity has been attributed to several mechanisms, including direct disruption of cell membranes, oxidative stress caused by reactive oxygen species generation, and interactions with intracellular components such as proteins and DNA (Yang et al., 2013a; Hu and Zhou, 2013). In addition, graphene materials may induce inflammatory responses by activating immune cells. Therefore, understanding how graphene-based nanomaterials interact with the immune system is crucial for evaluating their biosafety and long-term biomedical applications.

The innate immune system serves as the first line of defense against foreign materials and invading pathogens. Neutrophils are polymorphonuclear granulocytes that play a central role in innate immunity and are typically the first immune cells recruited to sites of infection or tissue injury. These cells eliminate pathogens through multiple mechanisms, including phagocytosis, degranulation, and the release of neutrophil extracellular traps (NETs). NETs are structures composed primarily of chromatin fibers decorated with antimicrobial proteins such as myeloperoxidase (MPO) and neutrophil elastase. These structures trap and neutralize extracellular pathogens in a process known as NETosis. Although NETosis is commonly associated with cell death, emerging evidence suggests that neutrophils may retain certain functional activities, such as chemotaxis and phagocytosis, even after NET release (Yipp et al., 2012). Recent studies have shown that primary human neutrophils can recognize GO in a size-dependent manner and respond by releasing NETs that contribute to the degradation of graphene-based materials (Kurapati et al., 2015; Mukherjee et al., 2018a; Mukherjee et al., 2018b). Furthermore, enzymatic degradation of carbon nanomaterials, including carbon nanotubes and GO, has been demonstrated through the action of peroxidases released by activated immune cells such as neutrophils and eosinophils (Kurapati et al., 2015; Mukherjee et al., 2018a; Russier et al., 2011; Andón et al., 2013).

Because of the potential toxicity of pristine GO, surface functionalization with biocompatible polymers has been widely explored as an effective strategy to improve the physicochemical and biological properties of graphene-based nanomaterials (Jiang et al., 2021; Qu et al., 2018; Zhang et al., 2017). Among these polymers, polyethylene glycol (PEG) is one of the most extensively used materials for improving the stability, dispersibility, and biocompatibility of nanomaterials in biological environments (Zhang et al., 2017). Another commonly used polymer is polyethyleneimine (PEI), a positively charged polymer that is widely used in gene delivery systems due to its strong interaction with negatively charged DNA and cell membranes (Liu et al., 2018). However, although PEI can enhance cellular uptake and gene transfection efficiency, its positive charge may also influence interactions with immune cells and biological molecules.

Although previous studies have investigated the toxicity and biodegradation of pristine graphene oxide, the effects of polymer functionalization on immune-mediated degradation of GO remain poorly understood. In particular, how PEG and PEI surface modifications influence the interaction of GO with neutrophil-derived components such as NETs and MPO has not been fully explored.

In this study, we functionalized GO with PEG and PEI and investigated the interactions of these materials with granulocyte-like cells derived from HL60 cells. Specifically, we examined cell viability in the presence of GO, GO-PEG, and GO-PEI and evaluated the biodegradation of these materials by granulocyte-like cells, NETs released from these cells, and MPO, a key enzyme present in NETs. We hypothesized that functionalization of GO with PEG or PEI would enhance its biocompatibility while altering its susceptibility to immune-mediated oxidative degradation. Understanding these interactions is essential for evaluating the safety and biomedical potential of functionalized graphene-based nanomaterials.

## 2 Materials and methods

### 2.1 Fabrication and characterization

#### 2.1.1 Materials

Highly concentrated single-layer GO was purchased from Graphene Supermarket. Poly(ethylene glycol) bis(3-aminopropyl)-terminated (NH_2_-PEG-NH_2_), 1-ethyl-3-(3-dimethylaminopropyl) carbodiimide (EDC), branched PEI, RPMI 1640 medium, penicillin, streptomycin, N,N-dimethylformamide (DMF), phorbol 12-myristate 13-acetate (PMA), myeloperoxidase (MPO), phosphate-buffered saline (PBS, 1×), (3-aminopropyl) triethoxysilane (APTES), diethylenetriaminepentaacetic acid (DTPA), and methanol were purchased from Sigma. Fetal bovine serum (FBS) was obtained from Gibco (Thermo Fisher Scientific). The alamarBlue™ cell viability reagent was purchased from Thermo Scientific (Thermo Fisher Scientific). All reagents were of analytical grade and used as received without further purification.

#### 2.1.2 Synthesis of GO-PEG

To synthesize GO-PEG, we followed the method developed by Zhang et al. (2016). Initially, we prepared 10 mL of 2 mg/mL GO using a highly concentrated single-layer GO. The solution was sonicated for 2 h at room temperature (power 100 kHz, frequency 30%). Subsequently, we added 0.01 g of poly (ethylene glycol) bis(3-aminopropyl)-terminated (NH*2*-PEG-NH*2*), 10 mM N-hydroxysuccinimide (NHS), and 8 mM EDC into the solution and stirred it at room temperature for 24 h. We dialyzed the mixture against Milli-Q water for 6 days, and the resulting mixture was washing several times using Milli-Q water. Finally, the product was diluted in 10 mL of Milli-Q water.

#### 2.1.3 Synthesis of GO-PEI

We used sonicated GO as the starting material as we did in the previous section. We conjugated GO with branched PEI through the formation of an amide bond in the presence of EDC (Zhang et al., 2011). To this, we added an aqueous solution of branched PEI (100 mg) and EDC solution (10 mg/mL, 5 mL). After stirring this mixture for 30 min, we again added an EDC solution (10 mg/mL, 15 mL) and continued stirring overnight. We dialyzed the mixture against Milli-Q water for a total of 6 days, and the resulting mixture was washed several times using Milli-Q water. Finally, the product was diluted in 10 mL of Milli-Q water.

#### 2.1.4 Characterization

The sizes of GO, GO-PEG, and GO-PEI particles were analyzed using dynamic light scattering (DLS) on a Zetasizer Advance Series Basic model (Malvern Panalytical Ltd., Malvern, United Kingdom) at room temperature. Measurements were conducted in plastic cuvettes with sample concentrations ranging from 20 to 40 μg/mL. The refractive index and viscosity of the water used as the dispersant were 1.330 and 0.8872 centipoise, respectively. The zeta potential of GO, GO-PEG, and GO-PEI was also measured on the Zetasizer at room temperature at a concentration of 20–40 μg/mL.

The samples were freeze-dried overnight (vacuum: 1.030 mbar). Infrared spectra were obtained using a Bruker Alpha Fourier-transform infrared (FTIR) spectrometer equipped with an attenuated total reflection (ATR) accessory featuring a diamond crystal. The measurements were performed at a resolution of 4 cm^−1^ with a scan duration of 16 s, similar to previously reported FTIR characterization procedures (Mohammed et al., 2022a; Mohammed et al., 2022b).

Raman spectroscopy was conducted using a WITec alpha300R Raman microscope (Ulm, Germany) with a ×100 objective, employing a 532-nm laser and a 600-g/mm grating. Spectra were collected over the range of 500–3,000 cm^−1^, with 10 accumulations and an integration time of 0.5 s per spectrum. For Raman mapping, we used the Raman spectra of 450 points located in 1 µm distance from each other in a rectangular area of 30 µm × 15 µm.

The freshly cleaved mica surface was incubated with 10 µL of 0.5% (V/V) APTES for 1 min. Then, the mica surface was rinsed five times with 1 mL of Milli-Q water and dried under a gentle stream of nitrogen gas. Atomic force microscopy (AFM) samples at 20 μg/mL were prepared by depositing 10 µL of solution onto the functionalized mica surface, followed by a 15-min incubation period to allow GO, GO-PEG, and GO-PEI settle to the surface. The mica surfaces were thereafter rinsed five times with 1 mL of Milli-Q water and dried under a gentle stream of nitrogen gas. Images were obtained using an NTEGRA Prima (NT-MDT, Moscow, Russia) setup equipped with a gold-coated single crystal silicon cantilever (NT-MDT, NSG01), with a resonance frequency of 150 kHz in tapping mode. Images were recorded in 512 × 512 pixels, at a scan rate of 0.5 Hz, and then processed using the Gwyddion software package 2.58 (http://gwyddion.net/download/2.58/): polynomial background subtraction followed by planar subtraction and adjustment of horizontal aberrations. Individual length was measured manually, while the height profile was measured perpendicularly to the axis, and the associated mica background was subtracted.

### 2.2 Cell culture

All tests were conducted using HL60 cells. The HL60 cells were generously provided by Prof. Alexandra Stubelius from the Chalmers University of Technology. The cells were confirmed to be mycoplasma-free by PCR testing. All cell cultures were maintained at a temperature of 37 °C with 5% CO_2_ in RPMI medium supplemented with 10% fetal bovine serum (Gibco) (Breitman et al., 1980).

### 2.3 Viability assay using the alamarBlue assay

Cells were plated onto 96-well plates at a density of 2 × 10^4^ cells per well and cultured for 24 h before treatment. Then, the cells were treated with 400 μg/mL of GO, GO-PEI, and GO-PEG for 24 h. Along with addition of materials to the medium, we also added 100 units per milliliter (U/mL) of penicillin and 100 μg/mL of streptomycin to the culture medium. Then, the cells were incubated with a medium containing 1× alamarBlue (Thermo Scientific) staining solution for 3 h. The signal from the cells was detected using a FLUOStar Omega microplate reader, and the results were normalized to the medium control.

### 2.4 Cell treatment with GO and GO-functionalized derivatives for Raman measurements

HL60 cells were plated onto 48-well plates at a density of 5 × 10^4^ cells per well and cultured for 24 h. Then, the cells were treated with 400 μg/mL of GO, GO-PEG, and GO-PEI for 24 h. The materials from each well were collected and washed with Dulbecco’s phosphate buffered saline (DPBS) by centrifugation at 13,000 rpm. Then, 10 µL of samples was loaded onto the glass cover slide for Raman measurements.

### 2.5 NET induction

To induce differentiation in HL60 cells, HL60 cells were seeded in a T-75 cell culture flask and incubated in the presence of 70 mM DMF for 5 days in 37 °C with 5% CO_2_ (Najmeh et al., 2015). The medium in the flask was replaced with the fresh media containing the same concentration of the DMF after 2–3 days. Then, the differentiated cells were stimulated for NET formation by PMA. PMA at the concentration of 2 mM was already dissolved in DMSO and stored at −80 °C. We treated 2 × 10^8^ cells with 500 nM PMA in a Petri dish and then incubated for 4 h at 37 °C (to have enough NET, three Petri dishes were prepared). After 4 h, the medium was removed, and NETs formed on the bottom surface of Petri dishes were washed with cold PBS and collected for centrifugation at 450 *g* for 10 min at 4 °C. The supernatant was collected and divided in 1.5-mL tubes for centrifugation at 18,000 g for 10 min at 4 °C, and then, the DNA concentration was measured using NanoDrop. The concentration of DNA was 46.6 ng/μL.

### 2.6 Graphene oxide derivative biodegradation using NETs

The NET, obtained as mentioned in the previous section, was centrifuged at 18,000 *g* 4 °C for 10 min and further diluted in NaCl (140 mM) to the concentration of approximately 20 ng/μL (with respect to DNA content) for the biodegradation experiment (Mukherjee et al., 2018a). A 10-μL aliquot of GO, GO-PEG, and GO-PEI materials was loaded and dried on the glass cover slide. To study the biodegradation of GO and GO-functionalized derivatives using NETs, the freshly prepared NETs were loaded on the materials on the cover slide for 6 h at room temperature. H_2_O_2_ was charged to the samples at 25 µM every hour. The samples were briefly rinsed with Milli-Q water after 48 h and dried before Raman measurements. The samples without addition of NET were used as the control. To assess the effect of H_2_O_2_ alone, we also incubated the materials with H_2_O_2_ only.

### 2.7 MPO treatment

We first diluted the enzyme to 4 μg/μL using the buffer (100 mM NaCl and 50 mM sodium acetate, pH 6.0), according to the manufacturer’s protocol. Then, the MPOs were suspended in reaction buffer (50 × 10^−3^ M phosphate buffer containing 140 × 10^−3^ M NaCl and 100 × 10^−6^ M DTPA) (Martín et al., 2019). A 10-μL aliquot of GO, GO-PEG, and GO-PEI materials was loaded and dried on the glass cover slide. The reaction mixture was loaded on the materials on the cover slide and incubated at 37 °C for 6 h. H_2_O_2_ was added at a dose of 0.2 mM per hour. The samples were briefly rinsed with Milli-Q water after 48 h and dried before Raman measurements. The samples without addition of MPO were used as the control. To assess the effect of H_2_O_2_ alone, we also incubated the materials with H_2_O_2_ only.

### 2.8 HL60 differentiation microscopy observation

For differentiation, 50,000 HL60 cells were seeded in a T-25 cell culture flask and incubated in the presence of 70 mM DMF for 5 days in 37 °C with 5% CO_2._ Based on a previous study by Manda-Handzlik et al. (2018), DMF is suggested as the best stimulus for HL60 cell differentiation for NET studies (Manda‐Handzlik et al., 2018). The medium in the flask was replaced with the fresh media containing the same concentration of DMF after 3 days. For the control, cells were grown normally in the medium without any DMF. After 5 days of culture, the cells were collected through centrifugation at 250 *g* for 5 min and resuspended in the medium. The cells were smeared on the glass slide, fixed with 100% methanol for 5 min, air-dried, and further stained with May-Grünwald′s eosine–methylene blue solution modified. The staining procedure includes the following: 3-min staining with the staining solution, 6-min washing with 1× PBS, rinsing with PBS, and further washing with water. The slides were observed using a ×40 objective in brightfield mode with a Leica CTR4000 inverted microscope.

### 2.9 Treatment of granulocyte-like cells with GO and GO-functionalized derivatives

To induce the differentiation in HL60 cells, HL60 cells were seeded in a T-75 cell culture flask and incubated in the presence of 70 mM DMF for 5 days in 37 °C with 5% CO_2_. The medium in the flask was replaced with the fresh media containing the same concentration of the DMF after 2–3 days. Then, the granulocyte-like cells were collected and washed with PBS. A total of 8 × 10^4^ cells were cultured in T-25 cell culture flasks and treated with the culture medium supplemented with 200 μg/mL of GO, GO-PEG, and GO-PEI materials, along with and 100 U/mL of penicillin and 100 μg/mL of streptomycin for 3 days. The medium only with GO, GO-PEG, and GO-PEI materials was used as the background. After 3 days, 100 µL of the culture was incubated with 1× alamarBlue staining solution at 37 °C for 3 h. The signal from the cells was detected using the FLUOStar Omega, and the results were normalized to the medium control. The GO, GO-PEG, and GO-PEI materials were collected and washed with Milli-Q water several times, diluted with the same volume of water used for treatment, and loaded on the cover slide for Raman measurements.

### 2.10 Transmission electron microscopy (TEM)

A total of 3.7 × 10^7^ cells were added to T-75 flasks supplemented with 70 mM concentration of DMF for 5 days. After 2–3 days, the new medium plus DMF was supplemented to the culture. Then, the cells were collected and washed with PBS. A total of 3.7 × 10^7^ cells were treated with 200 μg/mL graphene oxide materials for 24 h before transmission electron microscopy (TEM). The samples were then stained with 1% osmium tetroxide and 1% aqueous uranyl acetate in 0.1 M Na-cacodylate buffer. After staining, samples were subjected to dehydration series with ethanol (30%, 50%, 70%, 85%, 95%, and 100%) and infiltration series with epoxy hard plus resin (25%, 50%, 75%, and 100%). Finally, the samples were polymerized in BEEM capsules at 60 °C for 16 h. Ultrathin sections (70 nm) were obtained using a Leica EM UC6 ultramicrotome and imaged using a Thermo Scientific Talos L120C transmission electron microscope.

### 2.11 Circular dichroism (CD)

Circular dichroism (CD) was used to assess the secondary structure of MPO in the absence and presence of the GO materials. MPO was diluted to 4 μg/μL in 100 mM NaCl and 50 mM sodium acetate buffer (pH 6.0), according to the manufacturer’s preparation method. A volume of 5 μL of the MPO solution was then suspended in reaction buffer (50 × 10^−3^ M phosphate buffer containing 140 × 10^−3^ M NaCl and 100 × 10^−6^ M DTPA), and 1 µL of GO, GO-PEG, and GO-PEI materials was added to the total volume of 20-µL reaction mixture. H_2_O_2_ was added at a dose of 0.2 mM per hour. The reaction mixture was incubated at 37 °C for 5 h. The reaction buffer was used as the background and subtracted from all recorded CD spectra. The CD measurements were performed using a Chirascan CD spectrometer (Applied Photophysics) from 280 to 190 nm using a quartz cuvette (Hellma Micro Absorption Cuvettes Z800015) with a path length of 0.1 cm, a bandwidth of 1 nm, a time per point of 0.5 s, and a step size of 0.2 nm. Three individual spectra were acquired and averaged for each condition before correcting the spectra for background contributions. All spectra were recorded at room temperature.

### 2.12 Molecular dynamics (MD) simulations

#### 2.12.1 Building the models

pG was constructed using the graphene builder plugin in VMD (Humphrey et al., 1996). To create molecular models of GO and GO functionalized with polyethylene glycol and polyethyleneimine, a Python script was employed in conjunction with the Atomic Simulation Environment (ASE) (Hjorth Larsen et al., 2017). The GO sheets were derived from a pG layer measuring 50 × 50 Å and involved the random addition of acid groups (−COOH), hydroxyl (−OH), and epoxide (−O) groups to both the top and bottom surfaces; additional COOH groups were also positioned at the edges. The resulting functional group ratios were defined as C_10_O_0.5_(OH)_1_(COOH)_1_, indicating that for every 100 carbon atoms in pristine graphene, there are five epoxy groups, 10 hydroxyl groups, and 10 carboxyl groups. This modification yields a degree of oxidation of approximately 25%, calculated as the ratio of oxygen-containing groups to carbon atoms. This methodology is consistent with approaches used in other studies (Mkhoyan et al., 2009; Mahdavi et al., 2020). After generating GO, we prepared two functionalized structures: GO with polyethylene glycol (GO-PEG) and GO with polyethyleneimine (GO-PEI). The polymer chains were covalently linked to the carboxyl groups on both the edges and the basal plane through amide bond formation, enhancing the materials’ compatibility and functionality. This dual functionalization strategy improves biocompatibility and facilitates tailored interactions with various biological systems and applications (Dziewięcka et al., 2021; Lehner et al., 2021; Jana et al., 2013; Xu et al., 2014).

#### 2.12.2 System preparation

The molecular system, comprising GO alone and that functionalized with PEG and PEI groups, was prepared using LigPrep in Schrödinger Maestro (version 2024-1; www.schrodinger.com). System Builder was then employed to solvate the functionalized GO in an orthorhombic simulation box, with box dimensions chosen to provide at least a 10-Å solvent buffer around the solute. TIP3P water molecules (Jorgensen et al., 1983) were added to replicate an aqueous environment, and Na^+^ and Cl^−^ ions were added to neutralize the system and to yield a final NaCl concentration of 150 mM. After solvating the system, the configuration was validated to ensure that there were no steric clashes or overlaps between components. A short energy minimization step was performed to refine the initial setup, and the optimized system was exported as the input for multistage molecular dynamics equilibration, ensuring that the orthorhombic box parameters and periodic boundary conditions were preserved for subsequent simulations.

#### 2.12.3 System equilibration

For the multistage equilibration of the GO and functionalized GO systems, the Desmond program (Bowers et al., 2006) in Schrödinger Maestro (version 2024-1) was used.

The equilibration process began with a 500-ps Brownian dynamics simulation at 10 K to resolve any initial steric clashes and optimize the molecular packing. Following this, the system underwent a 0.1-ns equilibration in the NVT ensemble at 303.15 K. During this stage, position restraints were applied to the heavy atoms of the GO sheet and the attached functional groups using a force constant of 0.239 kcal/mol, with a timestep of 1 fs. This step allowed the water molecules and ions to adjust around the solute while maintaining the structural integrity of GO and its functionalized derivatives.

Subsequent equilibration stages involved simulations in the NPT ensemble to stabilize the system’s pressure and density under semi-isotropic pressure coupling. For adjusting the temperature and pressure of the systems, the Nose Hoover thermostat and the Martyna–Tobias–Klein barostat were employed at 300 K and 1.01325 bar, respectively (Martyna et al., 1992; Wentzco and vitch, 1991). The first NPT stage, lasting 5 ns, retained position restraints on the GO carbons, with the force constant reduced to 0.0239 kcal/mol Å, allowing for gradual relaxation of the system. A second 5-ns NPT simulation was then performed, with further reduced restraints (0.0048 kcal/mol) and an increased timestep of 2 fs. These steps allowed for the controlled relaxation of the GO structure while enabling the surrounding solvent and ions to equilibrate under near-physiological conditions.

Finally, the system was subjected to a 50-ns unrestrained NPT simulation at 303.15 K and 1 atm. This production-level equilibration allowed all components of the system, including the GO, functional groups, water molecules, and ions, to interact dynamically and reach a state of thermodynamic equilibrium. Throughout these stages, the system’s properties, such as root mean square deviation (RMSD), and energy fluctuations, were monitored to confirm convergence and stability.

This multistage equilibration protocol provided a robust foundation for further molecular dynamics simulations, ensuring that the system accurately represented the behavior of functionalized GO in an aqueous environment, suitable for subsequent adsorption or interaction studies.

#### 2.12.4 Disordered System Builder

The Disordered System Builder module in Schrödinger Maestro (version 2024-1) was utilized to prepare new multi-stage molecular system processing. The equilibrated GO model from the previous equilibration was used as the substrate. For the components, hypochlorous acid (HOCl) was introduced into the system at 10% concentration in combination with 90% water to create a solvent mixture. The aim was to investigate the adsorption behavior and interfacial interactions of HOCl at the GO surface and compare it with the conjugated counterparts.

An amorphous solvent environment was generated, ensuring that the solvent mixture was homogeneously distributed in the simulation box, which contained ∼22,500 atoms. The amorphous state was chosen to avoid crystallization effects and to mimic a realistic, disordered aqueous solution environment. The substrate type for the equilibrated GO model was a planar interface, and periodic boundary conditions were selected. The OPLS4 force field (Lu et al., 2021) was applied for all components in the system, including the GO and solvent components. This provided accurate modeling of the system’s molecular interactions and behavior. After ensuring that HOCl and water molecules were properly solvated and that no steric overlaps existed, the system underwent preparatory equilibration. The use of the Disordered System Builder facilitated the rapid creation of this solvent mixture and amorphous system, setting the stage for further molecular dynamics simulations. The prepared system was then subjected to multistage molecular dynamics simulations.

#### 2.12.5 MD multistage workflow

Brownian dynamics simulations were performed to further investigate the structural behavior and system stability of the functionalized GO system under controlled thermal conditions. A 100-ns Brownian dynamics simulation was conducted at 10 K to ensure the system’s stability, validate the minimization steps, and assess how the system behaves under low-temperature conditions. MD simulations were performed as the second stages. This step was essential to study the dynamic behavior of the system and the energy calculations at the GO–HOCl–water interface. The MD multistage workflow was configured for a total simulation time of 20 ns in the NPT ensemble under constant temperature and pressure conditions. The system temperature was adjusted to 303.15 K, representing physiological conditions to mimic the realistic aqueous behavior.

#### 2.12.6 Analyses

To analyze the interactions between GO and HOCl in the simulated system, trajectory analysis and energy plots were employed. These analyses were based on data obtained from the 20-ns molecular dynamics simulations. We compared the adsorption and interaction behavior of HOCl with GO only and in the GO-PEG and GO-PEI forms. The comparison allowed us to evaluate how the functionalization of GO with PEG or PEI affected the adsorption mechanisms, structural stability, and interaction energetics of HOCl at the GO interface.

## 3 Results and discussion

### 3.1 Characterization of GO-PEG and GO-PEI

After the functionalization of GO with PEG and PEI, an increase in particle size was observed. The size of GO was initially approximately 1.5 µm, which changed to approximately 3.7 µm in GO-PEG and 1.7 µm in GO-PEI. The surface charge of GO was measured to be −59.8 ± 2.3 mV, and it was slightly reduced in GO-PEG (−31.2 ± 0.8 mV). As expected, GO-PEI showed a positive surface charge (24.9 ± 3.9 mV, Table 1).

**TABLE 1:** ID/IG ratios for various treatments.

Material	ID	IG	ID/IG
GO	∼114–119	∼113–117	∼1.01–1.02
GO MPO	∼16–26	∼16–25	∼1–1.04
GO H_2_O_2_	∼63–80	∼61–78	∼1.03
GO-PEG	∼126–133	∼119–126	∼1.05–1.06
GO-PEG MPO	∼92–109	∼88–104	∼1.04–1.05
GO-PEG H_2_O_2_	∼250–281	∼207–238	∼1.18–1.21
GO-PEI	∼228–249	∼194–215	∼1.16–1.17
GO-PEI MPO	∼120–131	∼104–113	∼1.15–1.16
GO-PEI H_2_O_2_	∼354–399	∼289–327	∼1.03

**FIGURE 1:**
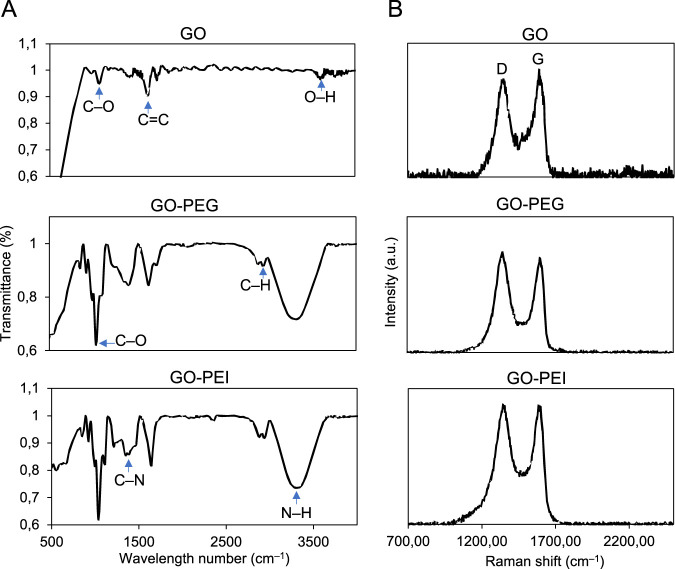
Characterization of graphene oxide derivatives. **(A)** FTIR analysis of GO, GO-PEG, and GO-PEI. The respective peaks are marked by blue arrows. **(B)** Raman spectra of GO, GO-PEG, and GO-PEI obtained through Raman microscopy.

In addition to the physical parameters such as size and surface charge, the chemical composition of the GO and its functionalized derivatives was characterized using FTIR and Raman spectroscopy (Figure 1). In FTIR analysis of GO, peaks at 1,665–1,760 cm^−1^ and 1,000–1,320 cm^−1^ demonstrated that GO possessed C=O and C–O groups, respectively. In the case of GO-PEG and GO-PEI, the peaks at 3,250–3,400 cm^−1^ and 665–910 cm^−1^ were assigned to N–H vibrations that were not found in GO samples. The peak at 1,020–1,250 cm^−1^ indicated C–N vibration in the PEG and PEI grafting in GO-PEG and GO-PEI (Choi et al., 2016).

Characteristic Raman peaks of graphene were detected in GO, GO-PEG, and GO-PEI samples. In Raman spectroscopy of graphene-based materials, two characteristic bands are commonly observed: the G band (∼1,580–1,590 cm^−1^) and the D band (∼1,320–1,350 cm^−1^) (Ferrari and Basko, 2013). The G band originates from the in-plane vibrational mode of sp^2^-hybridized carbon atoms in the hexagonal lattice and reflects the structural order of the graphitic network, whereas the D band arises from a defect-activated breathing mode of sp^2^ carbon rings and becomes prominent in the presence of structural defects such as edges, vacancies, or functional groups (Ferrari and Basko, 2013).

In addition, AFM was used to investigate the surface morphology and thickness of GO and its functionalized derivatives (GO-PEG and GO-PEI) (Supplementary Figure S1). The AFM image of pristine GO reveals thin sheet-like structures with irregular lateral morphology. The corresponding height profile measured across a representative flake indicates a thickness in the nanometer range, consistent with few-layer graphene oxide sheets. For GO-PEG, the AFM image shows more aggregated structures compared with pristine GO. The height profile across the selected region exhibits a broader and slightly multipeak distribution, suggesting the presence of stacked or clustered nanosheets. This behavior is likely associated with PEG chains attached to the GO surface, which can promote steric interactions and induce partial aggregation of the sheets. In the case of GO-PEI, the AFM image displays irregular clusters and rougher surface features compared with GO and GO-PEG. The corresponding height profile indicates a larger vertical variation, suggesting thicker aggregates or multilayer structures. This increase in surface roughness and thickness can be attributed to the presence of PEI, whose branched polymer structure may enhance intersheet interactions and promote the formation of compact clusters. Overall, the AFM results confirm that surface functionalization with PEG and PEI alters the morphology and thickness distribution of GO sheets, leading to increased aggregation and surface roughness compared with pristine GO.

### 3.2 Conjugation of PEG and PEI to GO makes the material more tolerable by the granulocyte-like cells

**FIGURE 2:**
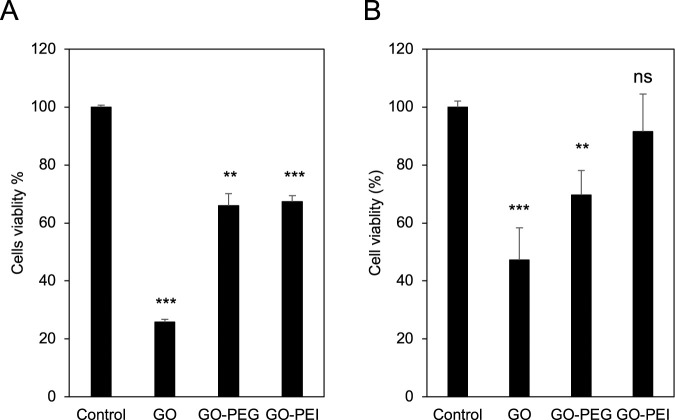
The viability of HL60 and granulocyte-like cells in the presence of GO, GO-PEG, and GO-PEI. **(A)** The HL60 cells were exposed to 400 μg/mL GO, GO-PEG, and GO-PEI for 24 h. **(B)** The HL60 cells were induced to be differentiated using 70 mM DMF, followed by exposure to 200 μg/mL GO, GO-PEG, and GO-PEI for 3 days. Relevant to **(A,B)**, the viability was measured through alamarBlue staining. The medium only with GO, GO-PEG, and GO-PEI materials was used as the background. The results were normalized to the control samples that were the cells growing under normal conditions. Data represent the mean ± SE of three independent replicates, and it was statistically analyzed and compared with the control (ns: not significant, *P < 0.05, **P < 0.01, and ***P < 0.001) using Student’s t-test.

To demonstrate the effect of GO derivatives on HL60 cells, the impacts of GO, GO-PEG, and GO-PEI on cell viability were investigated. For this, HL60 cells were grown and treated with 400 μg/mL of GO, GO-PEG, and GO-PEI for 24 h. Then, the viability of the treated cells was measured using alamarBlue staining, and the results were normalized to the control samples, which were the cells growing normally in the culture medium without the test materials. The culture medium with the materials but without cells was used as the background. AlamarBlue staining is indicative of cell viability. As indicated in Figure 2A, GO-PEG and GO-PEI samples showed a higher number of alive cells compared to the GO alone. The number of alive cells was 2.5–2.6 times higher in the presence of GO-PEG and GO-PEI, compared to GO alone.

In addition to this, the effects of GO, GO-PEG, and GO-PEI on the viability of granulocyte-like cells (Figure 2B) were also examined. For this purpose, HL60 cells were first induced with 70 mM DMF for 5 days to differentiate into granulocyte-like cells. Following differentiation of HL60, the presence of granulocyte-like cells that were morphologically similar to mature granulocytes with characteristic band-like or multilobulated nuclei shape was confirmed through May-Grünwald′s eosine–methylene blue solution modified staining (Supplementary Figure S2) and bright-field microscopy (Supplementary Figure S3). These granulocyte-like cells were collected and treated with 200 μg/mL GO, GO-PEG, and GO-PEI for 3 days. The concentration of materials used to treat the differentiated cells was reduced to 200 μg/mL compared to that of 400 μg/mL for non-differentiated cells because the differentiated cells were treated for a longer duration of 3 days. After 3 days of treatment, 100 µL of the culture was collected and stained with 1× alamarBlue staining solution. The medium supplemented with GO, GO-PEG, and GO-PEI materials without cells was used as the background, and the results were normalized to the control cells growing under normal conditions in the absence of test materials. The alamarBlue assay demonstrated that 91.5% of cells were alive after GO-PEI treatment, while the GO-PEG biocompatibility was slightly weaker with 69.7% of cells being alive. However, only 47.3% of cells were alive after 3 days of treatment with GO. Moreover, the difference in cell viability between GO-PEG and GO-PEI was not statistically significant. Overall, the findings with granulocyte-like cells, differentiated from HL60 cells, were similar to results obtained with non-differentiated cells.

PEG is commonly considered a safe polymer since it has been approved by the United States Food and Drug Administration (FDA) for diverse applications in humans. PEG is the most well-studied polymer regarding the modification of GO nanomaterials for drug delivery. As reported by previous studies, PEG coatings contribute to stabilizing GO nanomaterials in physiological environments, promoting cellular uptake of GO, increasing drug loading/releasing behavior, and decreasing cytotoxicity (Liu et al., 2008; Xu et al., 2014; Chowdhury et al., 2015; Chu et al., 2018). A long-term study on the distribution and toxicology of PEGylated GO nanomaterials *in vivo* further demonstrated dramatic merits of such functionalization (Yang et al., 2011; Yang et al., 2013b). Our study also demonstrates that the conjugation with PEG decreased the cytotoxicity of GO toward undifferentiated and differentiated granulocyte-like cells.

PEI is a common polymer for nucleic acid compaction in non-viral delivery vectors and can contribute to high transfection efficiency in different cell lines (Pezzoli et al., 2012). However, PEI exhibits poor biocompatibility and has been associated with cytotoxicity, which has limited its clinical applications. However, a significant decrease in cytotoxicity was observed when PEI was grafted to the surface of GO (Wang et al., 2020). Furthermore, the cytotoxicity against MG63 or U2OS cell lines had no significant differences among different GO-PEI-treated groups (5–30 μg/mL). Mild toxicity was reported when the concentration of GO-PEI reached 40 or 50 μg/mL. The GO-PEI showed low cytotoxicity, even when the high-molecular-weight PEI (25 kDa) was used, suggesting that GO-PEI complexes were much safer than the single PEI polymers (Ou et al., 2020). The current study shows that the conjugation with PEI decreased the cytotoxicity of GO, which corroborates earlier reports mentioned above.

### 3.3 Interaction of granulocyte-like cells with GO and GO derivatives

**FIGURE 3:**
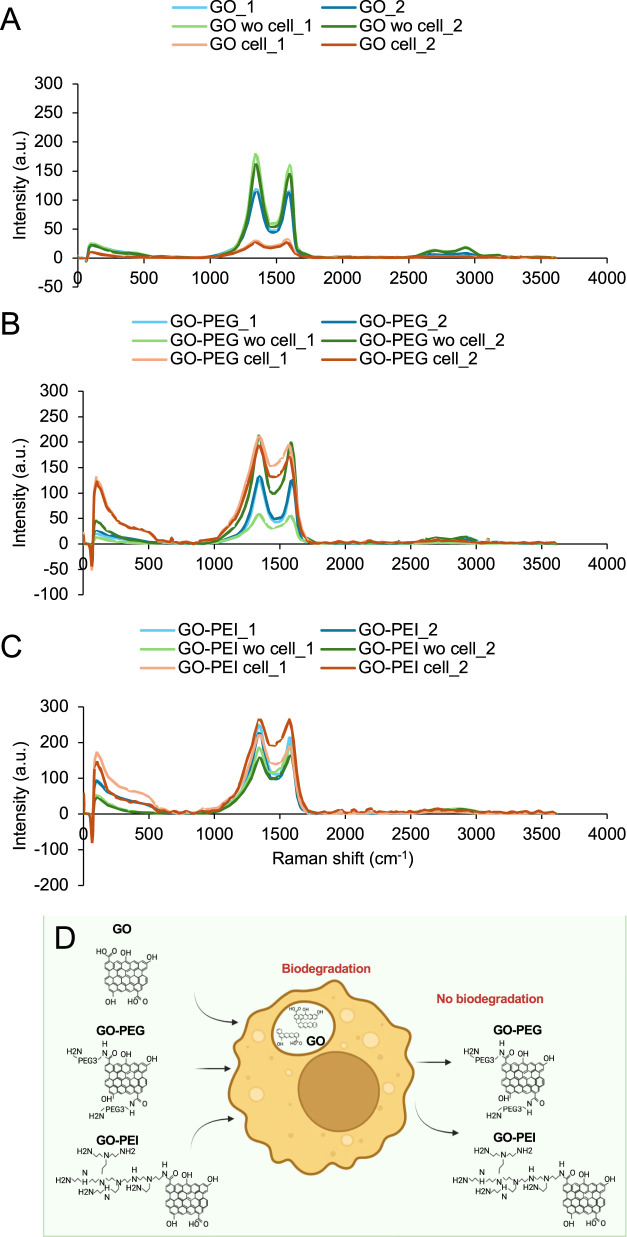
Granulocyte-like cells biodegrade GO but not GO-PEG and GO-PEI. Effect of granulocyte-like cells on the Raman spectra of **(A)** GO, **(B)** GO-PEG, and **(C)** GO-PEI (orange and red curves). Each graph represents the average Raman spectra of 450 points located in a 1 µm distance from each other in a rectangular area of 30 µm × 15 µm on GO, GO-PEG, and GO-PEI samples treated with granulocyte-like cells. Two different regions marked with _1 and _2 are shown. The control samples are GO, GO-PEG, and GO-PEI incubated in the culture medium without any cells marked with “wo cell” (light and dark green curves) and GO, GO-PEG, and GO-PEI without any cell and without any incubation in the culture medium (light and dark blue curves). **(D)** Schematic representation of GO biodegradation by granulocyte-like cells.

As previously reported, GO can undergo biodegradation (Kurapati et al., 2015; Mukherjee et al., 2018a; Mukherjee et al., 2018b). Therefore, the effect of granulocyte-like cells on GO, GO-PEG, and GO-PEI was investigated by analyzing Raman spectra of the materials following 3 days of incubation (200 μg/mL) with granulocyte-like cells. After incubation, the materials were collected and deposited onto the cover slide for Raman spectroscopy. A total of 450 points, located 1 µm apart ,within a rectangular area of 30 μm × 15 µm were analyzed in two different regions of each sample (marked _1 and _2) to assess potential structural changes in GO derivatives after exposure to the cells (Figure 3). The spectra obtained from cell-treated samples (orange and red curves) were compared with those of control samples consisting of GO, GO-PEG, and GO-PEI incubated in the culture medium without cells (light and dark green curves), as well as pristine materials without incubation (light and dark blue curves).

**FIGURE 4:**
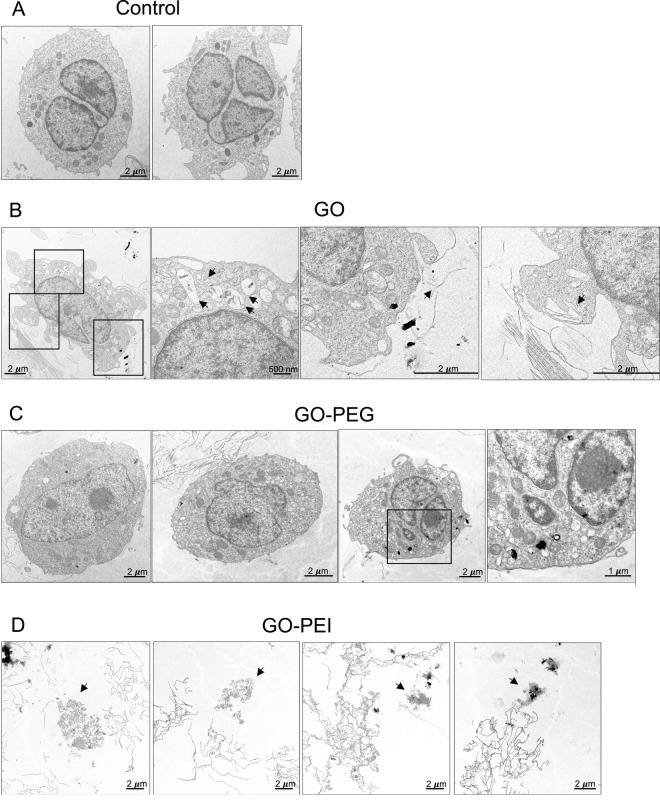
Interaction of granulocyte-like cells with GO, GO-PEG, and GO-PEI. **(A)** The cells were exposed to 0 μg/mL concentration of the material as the control and 200 μg/mL concentration of **(B)** GO, **(C)** GO-PEG, and **(D)** GO-PEI. TEM imaging was performed 24 h after exposure. Black arrows represent degranulation, NET release, and phagocytosis.

As shown in Figure 3A, GO samples incubated with granulocyte-like cells exhibited a noticeable reduction in Raman signal intensity compared with GO incubated without cells and pristine GO. The Raman intensity of GO incubated in the medium without cells was slightly higher than that of pristine GO, which may reflect the adsorption of medium components onto the GO surface during incubation. Although the reduction in the Raman signal observed in cell-treated samples may suggest structural modification or degradation of GO, it should be noted that changes in the Raman intensity can also arise from biomolecular adsorption, surface coverage by cellular components, or measurement artifacts. Therefore, Raman results alone cannot confirm the structural degradation of GO. However, when considered together with complementary observations from microscopy, the results support the possibility of cellular interactions contributing to GO modification (Figures 4A-D). Bright-field and TEM imaging revealed significant interactions between the materials and granulocyte-like cells (Supplementary Figures S4, S5). As shown in Figure 4B, degranulation was evident in the cells surrounded by GO sheets. Granules that carry cytotoxic molecules reach and fuse with the outer plasma membrane and finally release their content into the extracellular space (black arrows). Interestingly, the GO sheets in smaller sizes of approximately 260–850 nm could be entrapped within the cells through phagocytosis (black arrow). Similar to our observation, it was previously reported that the GO with 50–350 nm size was approximately engulfed 24%–34% more than GO with 750–1,300 nm (Ma et al., 2015). Thus, large-sized GO undergoes less cellular uptake compared to small-sized GO.

In addition to degranulation and phagocytosis, we could also observe anuclear cells that released NETs (Supplementary Figure S5A). The majority of DNA in NETs originates from the nucleus that produces them (Brinkmann et al., 1979). Thus, the release of nuclear DNA in this process followed by the rupture of the nuclear membrane (black arrow in Supplementary Figure S5A) resulted in anuclear cell morphology. The degranulation, phagocytosis, and NET release are known mechanisms by which neutrophil-like cells respond to foreign materials.

For GO-PEG and GO-PEI, the decrease in Raman intensity after exposure to cells was less pronounced (Figures 3B,C), suggesting that polymer functionalization may reduce the susceptibility of GO derivatives to structural modification or enzymatic attack under these conditions. Similar observations have been reported previously in studies investigating the degradation of carbon nanomaterials by peroxidase enzymes present in neutrophil extracellular traps (Mukherjee et al., 2018a; Martín et al., 2019; Kurapati et al., 2021). This observation aligns with those of recent studies demonstrating that surface functionalization of GO enhances its structural stability and modulates its interactions with biomolecules, thereby limiting direct contact with oxidative enzymes and reactive species (Pérez-Piñeiro et al., 2023; Angelopoulou et al., 2024). In particular, polymer coatings such as PEG introduce steric hindrance and increase hydrophilicity, thereby reducing surface reactivity and improving dispersion stability in biological environments (Bao et al., 2023).

TEM images revealed that the cells maintained a more normal morphology in interaction with GO-PEG and were less affected compared to GO as cellular compartments are still in place (Figure 4C; Supplementary Figure S5B). As the size of GO-PEG was larger than that of GO, there was no evidence of phagocytosis. However, similar to GO, degranulation could be found in the cells.

In case of GO-PEI, there was a significant interaction of GO-PEI with the cells as the remnant of cells could only be found in the GO-PEI-treated samples (Figure 4D; Supplementary Figure S5C). It is also possible that the fragile cells were also destroyed by the artifact of TEM. Nonetheless, the release of nuclear DNA from the cells was followed by the rupture of nuclear and plasma membranes, as shown by black arrows in Figure 4D. Previous studies have reported that GO significantly disrupts the lipid composition of neutrophil plasma membranes, leading to a reduction in cholesterol levels and an increase in oxidized cholesterol derivatives (Mukherjee et al., 2018b). However, there is no previous report on the interaction of GO-PEI and the neutrophil plasma membrane. Provided the positive surface charge of GO-PEI (Table 1), it is expected that there would be a significant interaction between GO-PEI and the net negatively charged cell membrane. However, despite the great interaction between the cells and GO-PEI, as verified by TEM, the Raman spectra intensity and degradation of GO-PEI were not affected after exposure to the cells.

Overall, the Raman data indicate that interactions between granulocyte-like cells and GO derivatives lead to detectable spectral changes, which may reflect the structural modification of GO. However, given the potential influence of biomolecular adsorption and experimental factors on Raman intensity, these results should be interpreted cautiously and in conjunction with complementary microscopy evidence demonstrating cellular processes such as phagocytosis, degranulation, and NET formation.

It is notable that the structural modification of GO was significant with granulocyte-like cells (Figure 3), but not with non-differentiated HL60 cells, as evidenced by Raman spectroscopy (Supplementary Figure S6). This suggests that only the differentiated cells with the ability to produce NETs are able to make structural modification in GO. Previous studies have shown that a bidirectional interaction between carbon nanotubes (CNTs) and immune cells. CNTs can initiate inflammatory responses by activating macrophages, neutrophils, and other immune cells. In turn, these inflammatory cells—particularly neutrophils—can degrade CNTs through enzymatic and oxidative mechanisms, thereby reducing their proinflammatory effects (Shvedova et al., 2012; Kagan et al., 2010). It can be suggested that the interaction of granulocyte-like cells with GO in the form of phagocytosis, degranulation, and NET release, followed by structural modification in GO, might help reduce the inflammatory potential of GO, and thus, these cells could survive under such conditions. However, the structural modification in GO did not result in enhanced growth of granulocyte-like cells and biocompatibility of GO compared with GO-PEG and GO-PEI. This is consistent with a previous report showing that GO induces loss of cell viability with the NET release (Mukherjee et al., 2018b).

Taken together with previous reports on MPO-mediated degradation of carbon nanomaterials and GO (Kurapati et al., 2015; Mukherjee et al., 2018a; Kurapati et al., 2021), our results suggest that granulocyte-like cells may contribute to the structural modification of GO through combined mechanisms including oxidative activity, NET release, and cellular interactions. However, given the limitations of Raman spectroscopy in distinguishing degradation from surface adsorption effects, these findings should be interpreted as indicative of potential structural modification rather than definitive evidence of complete biodegradation.

### 3.4 GO is degraded by NETs collected from granulocyte-like cells

**FIGURE 5:**
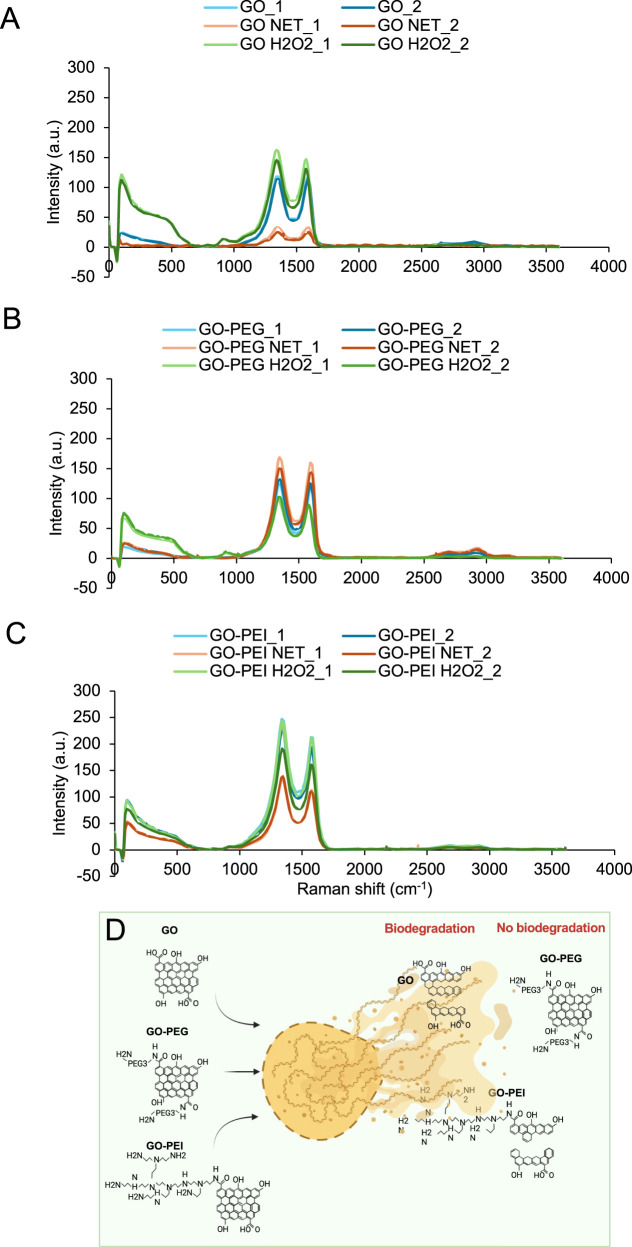
GO and GO-PEI are degraded by NETs collected from granulocyte-like cells. Effect of NETs on Raman spectra of **(A)** GO, **(B)** GO-PEG, and **(C)** GO-PEI. Each graph represents the average Raman spectra of 450 points located in 1 µm distance from each other in a rectangular area of 30 µm × 15 µm on GO, GO-PEG, and GO-PEI samples treated with NETs marked with “NET” (orange and red curves). Two different regions marked with _1 and _2 are shown. The control samples are GO, GO-PEG, and GO-PEI without NETs (light and dark blue curves). The GO, GO-PEG, and GO-PEI samples incubated with only H_2_O_2_ marked with “H_2_O_2_” (light and dark green curves). **(D)** Schematic representation of GO and GO-PEI biodegradation by NETs.

There are three strategies employed by neutrophils to eliminate attacking microbes: i) microbial uptake that is followed by intracellular destruction using oxidative and proteolytic enzymes, ii) degranulation that leads to the secretion of antimicrobial factors such as MPO and thereby extracellular destruction of microbes, and iii) NET release with entrapment and thus non-phagocytic killing of microbes (Papayannopoulos and Zychlinsky, 2009; Brinkmann et al., 1979). Huang et al. (2022) claimed that the micrometer-sized and nanometer-sized GOs selectively direct neutrophils to two distinct fates of NETosis by releasing NETs and degranulation, respectively (Huang et al., 2022). To explore whether NETs from granulocyte-like cells play a role in biodegradation of GO, as shown in our previous experiment (Figure 3A), HL60 cells were first induced to differentiate by DMF and then induced for NET formation using PMA treatment. The NETs formed from HL60 cells were collected and used to treat GO, GO-PEG, and GO-PEI loaded onto the cover slides (orange and red curves) (Figure 5). Since the neutrophils’ defensive behavior is accompanied with generation of reactive oxygen species and activation of p-ERK and p-Akt kinases (Huang et al., 2022), H_2_O_2_ was also supplied to the reaction mixture. After 48 h of incubation, the surface of the cover slides was rinsed with water and then dried for Raman mapping of the samples. We compared the results with the non-treated control samples of GO, GO-PEG, and GO-PEI samples (light and dark blue curves). It was previously reported that the pristine multilayered graphene can be degraded in the presence of H_2_O_2_ alone (Xing et al., 2014). This condition was, therefore, included as an additional control (light and dark green curves). The intensity of the characteristic Raman peak of GO samples was significantly reduced once GO is treated with NETs (Figure 5A). A similar effect of NETs was found in GO-PEI samples treated with NETs (Figure 5C). It is expected that the positively charged GO-PEI would be attracted to NETs whose backbone consists of DNA. H_2_O_2_ alone, at the relevant concentration used in this experiment, did not show any significant effect on the reduction in Raman peak intensity in any of the GO samples. These results suggest that the biodegradation of GO that was caused by granulocyte-like cells (Figure 3A) is most likely due to the NET activity toward GO.

### 3.5 MPO degrades GO and GO-PEI

**FIGURE 6:**
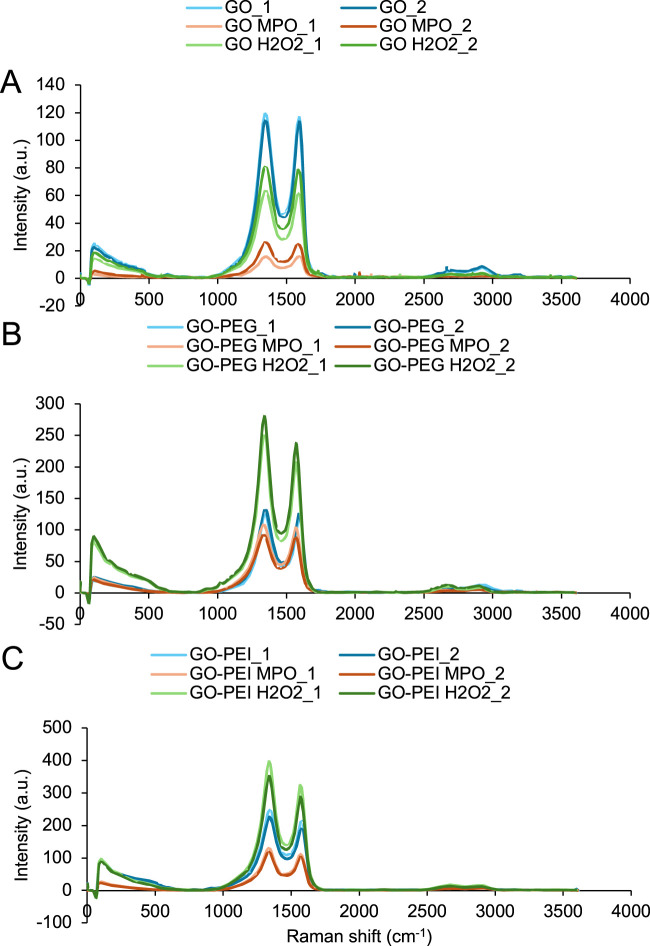
MPO degrades GO and GO-PEI. Effect of MPO on Raman spectra of **(A)** GO, **(B)** GO-PEG, and **(C)** GO-PEI. Each graph represents the average Raman spectra of 450 points located in 1 µm distance from each other in a rectangular area of 30 µm × 15 µm on GO, GO-PEG, and GO-PEI samples treated with MPO marked with “MPO” (orange and red curves). Two different regions marked with _1 and _2 are shown. The control samples are GO, GO-PEG, and GO-PEI without MPO (light and dark blue curves). The GO, GO-PEG, and GO-PEI samples incubated with only H_2_O_2_ marked with “H_2_O_2_” (light and dark green curves).

MPO is one of the key enzymes present in NETs (Kr et al., 2015). It catalyzes the production of HOCl from the reaction of H_2_O_2_ and chloride anions (Cl^−^) (Hampton et al., 1998). To explore whether MPO was involved in GO and GO-PEI biodegradation using NETs in the previous experiment (Figure 5), GO, GO-PEG, and GO-PEI solutions were loaded onto the glass slide, followed by the MPO reaction mixture including H_2_O_2_ being further loaded to the samples. The samples were rinsed with water, dried, and used for Raman mapping (Figure 6). Pristine GO, GO-PEG, and GO-PEI samples (light and dark blue curves) were used as controls. To determine potential biodegradation of materials by H_2_O_2_ itself, the GO, GO-PEG, and GO-PEI samples were also treated with H_2_O_2_ alone, the same concentration as we used for the MPO experiment (light and dark green curves). Compared to the previous section and the NET experiment, the concentration of H_2_O_2_ used for this experiment was 200 µM (Martín et al., 2019), which was higher than that used in the NET experiment (25 µM) (Mukherjee et al., 2018a). The Raman mapping showed that there was a significant decrease in the characteristic peak intensity of GO and GO-PEI samples subjected to MPO compared to that of the control GO and GO-PEI samples (Table 1; Figures 6A,C). This effect was specific to GO and GO-PEI samples as there were no significant differences between GO-PEG MPO and GO-PEG. H_2_O_2_ at the relevant concentration used in this experiment showed no significant effect on the reduction in Raman intensity and degradation, consistent with the control experiment in *Section 3.4*.

We measured the ID/IG ratios of GO materials upon treatment with H_2_O_2_ alone and MPO in the presence of H_2_O_2_ (Table 1). The ID/IG ratio of pristine GO was approximately 1.01–1.02, indicating a relatively balanced level of graphitic ordering and defect density typical for GO. After MPO treatment, the ID/IG ratio remained close to unity (∼1–1.04), suggesting that MPO alone causes only limited changes in the defect structure of GO. In contrast, incubation with H_2_O_2_ produced a slight increase in the ID/IG ratio (∼1.03), indicating mild oxidation-induced defect formation. Functionalization with polymers significantly influenced the Raman parameters. GO-PEG exhibited slightly higher ID/IG ratios (∼1.05–1.06) compared with GO, suggesting an additional structural disorder introduced by PEG functionalization. Notably, GO-PEG treated with H_2_O_2_ showed a pronounced increase in ID/IG (∼1.18–1.21), indicating a substantial increase in the defect density and disruption of the graphitic lattice under oxidative conditions. Similarly, GO-PEI displayed elevated ID/IG values (∼1.16–1.17) relative to GO, suggesting that PEI functionalization introduces additional structural defects. MPO treatment caused only minor changes in the ID/IG ratio (∼1.15–1.16), whereas exposure to H_2_O_2_ reduced the ratio (∼1.03), possibly reflecting oxidative modification and partial removal of disordered regions.

Analysis of the peak positions revealed slight shifts in both D and G bands depending on the treatment (Table 2). The D band shifted from ∼1344–1346 cm^−1^ in GO to lower values (∼1331–1339 cm^−1^) in functionalized materials and after MPO/H_2_O_2_ treatment, suggesting changes in the defect structure and local bonding environments. Similarly, the G band exhibited shifts from ∼1594 cm^−1^ in GO to lower values (∼1567–1577 cm^−1^) in GO-PEG and GO-PEI samples, which may indicate alterations in the sp^2^ domain size and electronic structure following functionalization and oxidative treatment.

**TABLE 2:** D and G peak positions for various treatments.

Material	D band (cm^−1^)	G band (cm^−1^)
GO	∼1,344–1346	∼1,594
GO MPO	∼1,344–1,349	∼1,586–1,589
GO H_2_O_2_	∼1,341	∼1,584–1,586
GO-PEG	∼1,344	∼1,591–1,596
GO-PEG MPO	∼1,331	∼1,567–1,569
GO-PEG H_2_O_2_	∼1,339	∼1,569–1,572
GO-PEI	∼1,341–1,344	∼1,577
GO-PEI MPO	∼1,336–1,339	∼1,569
GO-PEI H_2_O_2_	∼1,339	∼1,569

#### 3.5.1 Influence of GO, GO-PEG and GO-PEI on MPO secondary structure

**FIGURE 7:**
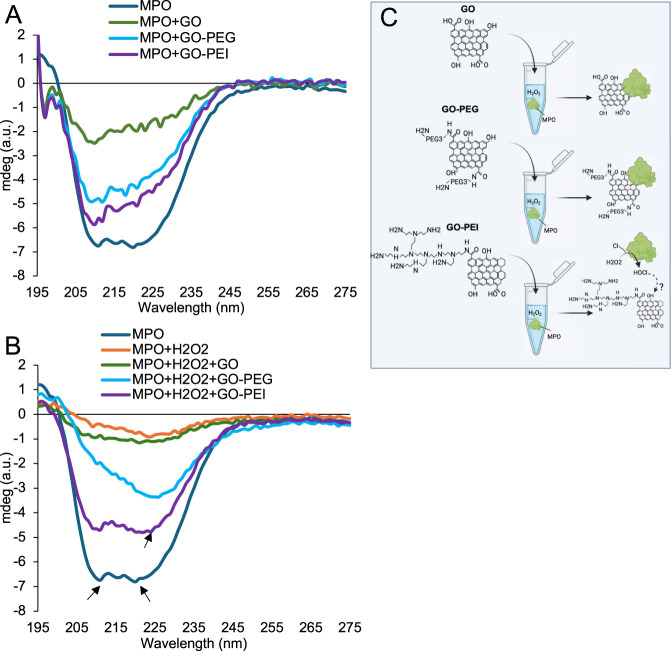
Circular dichroism analysis of MPO and GO, GO-PEG, and GO-PEI in the **(A)** absence and **(B)** presence of H_2_O_2_. Typical bands in α-helix and β-sheet structures are shown by arrows. **(C)** Schematic representation of the interaction of MPO with GO, GO-PEG, and GO-PEI.

A molecular docking study revealed that MPO has a single binding site for GO. Specifically, residues Asp321, Arg323, Ser19, and Arg31 engage with GO through electrostatic interactions, while Ile160 and Pro34 rely on hydrophobic interactions for binding to GO (Huang et al., 2022). To further elucidate the interactions of MPO with GO, GO-PEG, and GO-PEI, we conducted CD spectroscopy analysis to explore the secondary structure of MPO in the absence and presence of these compounds (Figures 7A-C). CD measurements were performed both with and without H_2_O_2_ (Figures 7A,B). CD was used to investigate whether GO derivatives influence the secondary structure of MPO. The CD spectrum of MPO shows two characteristic negative bands at approximately 208 nm and 222 nm, which are typical for α-helical protein structures. The presence of these two bands indicates that the α-helical conformation of MPO is largely maintained even after interaction with graphene-based materials (Figure 7A). However, noticeable differences in the signal are observed when MPO interacts with GO materials. In particular, the narrowing of the band near 222 nm suggests partial destabilization of the α-helical arrangement within the MPO structure. In addition, the overall reduction in the signal may indicate conformational rearrangements or partial unfolding of the protein. These changes are likely associated with the adsorption of MPO onto GO surfaces, potentially driven by electrostatic interactions between MPO and GO, as previously reported (Huang et al., 2022). Among the tested materials, unmodified GO produces the strongest decrease in the signal, indicating a more pronounced perturbation of MPO secondary structure. In contrast, GO-PEG and GO-PEI induce smaller spectral changes, suggesting that surface functionalization reduces the extent of structural perturbation. These observations indicate that PEG and PEI modifications partially preserve the native conformation of MPO, possibly by reducing direct interactions between the enzyme and the graphene surface.

Similar structural perturbations of proteins upon interaction with graphene-based materials have been reported, where adsorption onto graphene surfaces can induce conformational rearrangements depending on the surface chemistry and charge (Kumar and Parekh, 2020).

#### 3.5.2 Effect of H_2_O_2_ on MPO structural interactions with GO materials

The presence of H_2_O_2_ significantly influences MPO structure and its interaction with GO derivatives (Figure 7B). MPO is known to utilize H_2_O_2_ generated during the oxidative burst of neutrophils to produce a variety of antimicrobial oxidants (Paumann-Page et al., 2013). Consistent with this catalytic role, the addition of H_2_O_2_ results in changes in the CD spectrum of MPO, including a reduction in the intensity of the α-helical bands, suggesting structural rearrangements or partial destabilization of the helical conformation.

High-tension (HT) signal analysis further indicates a reduction in voltage for MPO in the presence of H_2_O_2_ (Supplementary Figure S7), which may suggest protein aggregation or precipitation under oxidative conditions.

The presence of GO derivatives modulates these effects. In particular, GO-PEG appears to enhance the structural perturbation of MPO under oxidative conditions, as indicated by a pronounced reduction in the signal at approximately 208 nm. This behavior may be related to the PEG chains, creating a more hydrophilic environment around MPO, which could alter protein–surface interactions and influence conformational stability.

Interestingly, MPO maintains its native structure to a greater extent in the presence of GO-PEI compared with unmodified GO when H_2_O_2_ is present. One possible explanation is related to the polycationic nature of MPO, which may reduce strong interactions with the positively charged GO-PEI surface. MPO has previously been shown to bind preferentially to negatively charged microbial surfaces, contributing to antimicrobial activity (Miyasaki et al., 1987).

#### 3.5.3 Implications for MPO-mediated degradation of GO materials

Beyond direct protein–material interactions, MPO catalyzes the formation of HOCl, a strong oxidant, through the reaction between H_2_O_2_ and Cl^−^. The oxidants produced during this process constantly attack the graphene nanoribbons, increasing surface defects and causing damage to the original clusters (Kotchey et al., 2012).

The relatively better preservation of MPO structure in the presence of GO-PEI may, therefore, be important for maintaining enzymatic activity, potentially contributing to the more efficient degradation of GO-PEI observed in this study.

### 3.6 Molecular dynamics simulation studies

#### 3.6.1 Model structures

To investigate the effect of HOCl, the catalytic products of the reaction between H_2_O_2_ and Cl^−^ by MPO, GO, GO-PEG and GO-PEI were used as starting points for the molecular dynamics simulations. The structural differences between the three model systems are illustrated in Supplementary Figure S8.

#### 3.6.2 System preparation and molecular dynamics simulations

The initial system representation used for MD simulations is shown in Supplementary Figure S9.

The system consists of the GO scaffold placed in the center of the simulation box, surrounded by a solvation layer (Supplementary Figure S9A). The red-colored components on the top of the box represent HOCl, which was introduced as the ligand of interest for the adsorption studies, and the green-colored components represent water. This initial representation provides the starting point for simulating the interactions between GO and HOCl. The two conjugated models (Supplementary Figures S9B,C) were solvated in the same simulation box setup with HOCl in a mixed aqueous environment.

**FIGURE 8:**
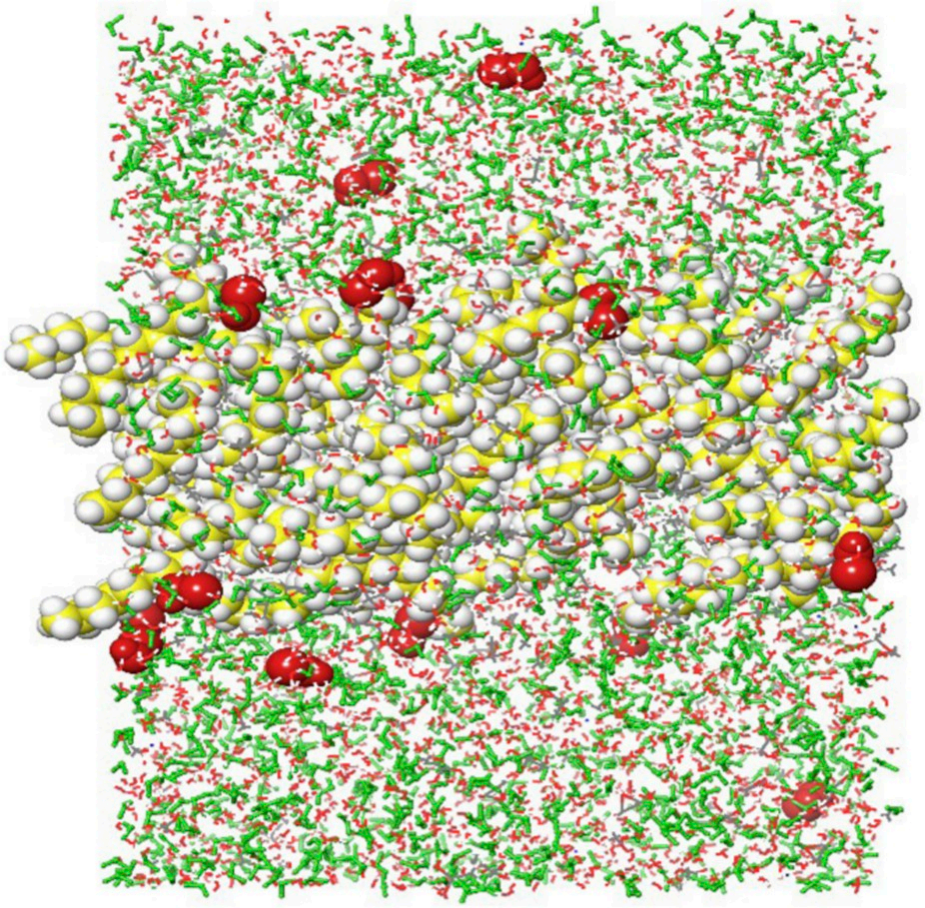
Interaction of HOCl (red) with the GO scaffold (yellow and white) from a snapshot after 11-ns MD simulations.

After the 20-ns MD simulations, the results indicated that HOCl successfully penetrated the GO scaffold, with both adsorption at the interface and subsequent diffusion into the internal regions of the GO structure, as shown in Figure 8. Similar results were observed for the conjugated GO-PEG and GO-PEI system, with some differences that are explained in detail in the next section.

#### 3.6.3 Trajectory density analyses

**FIGURE 9:**
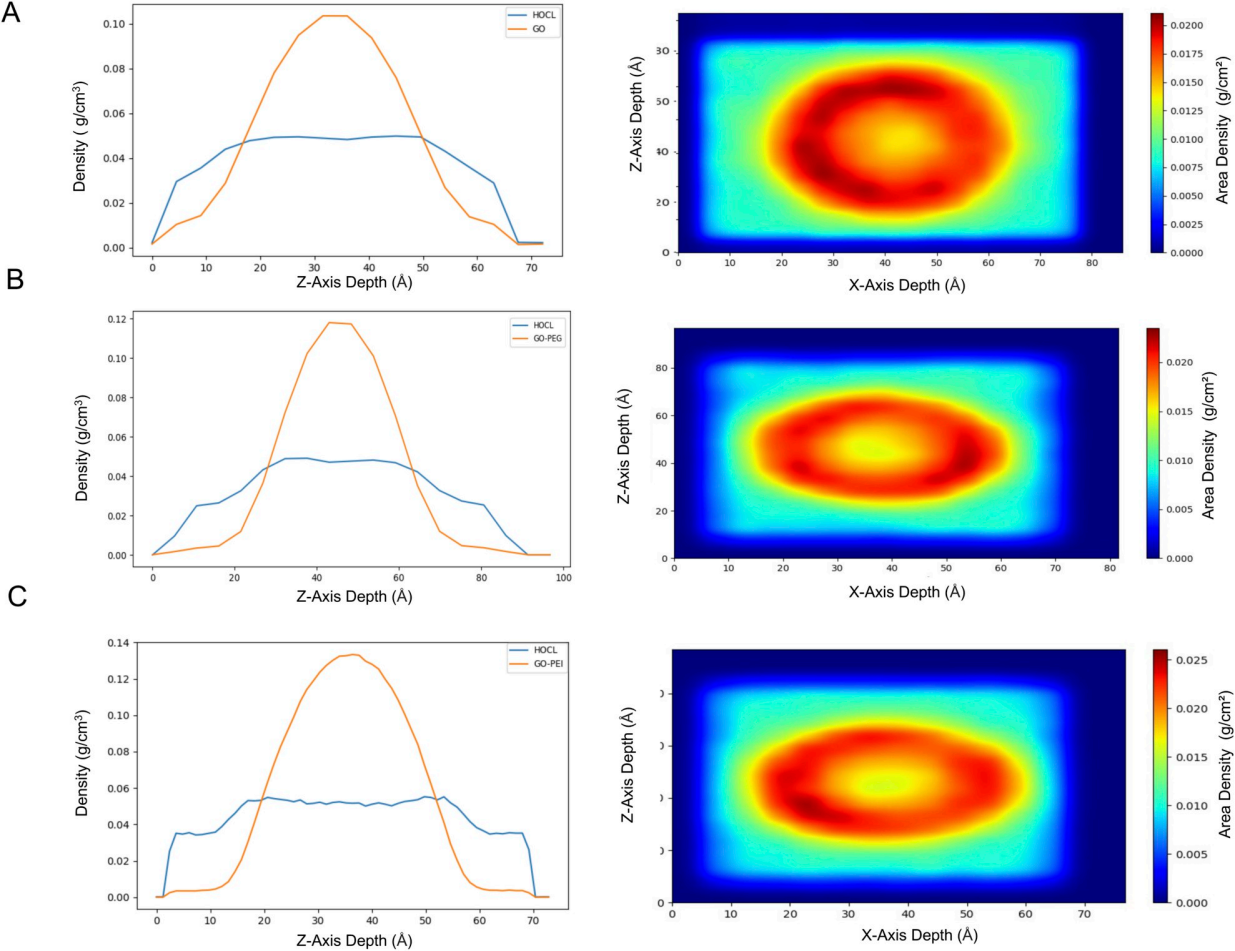
(Left) Density vs. Z-axis depth. The graphs show the density profiles of GO or its functionalized derivatives and the ligand (HOCl) as a function of Z-axis depth (in angstroms, Å). (Right) Area density heatmaps for HOCl. The heatmaps represent the 2D distribution of area density (in g/cm^2^) as a function of the X- and Z-axis depths for GO **(A)** and the functionalized derivatives GO-PEG **(B)** and GO-PEI **(C)**.

After the MD simulations, trajectory analyses were performed. Figure 9A shows graphs comparing the density profiles of the GO scaffold and HOCl ligands along the Z-axis, indicating the relative positions of GO and HOCl within the simulated box. The HOCl density is observed to overlap with the GO region, particularly from 15 Å to 50 Å, indicating that HOCl is entering and interacting with the GO scaffold, although not in a highly concentrated manner (moderate). The density gradually decreases outward, indicating that the GO scaffold’s density is localized, while any surrounding components (HOCl) are spread further out.

The results for GO-PEG (Figure 9B) indicate a significantly stronger retention of HOCl compared to pure GO, as evidenced by the higher peak density and the narrower peak profile. In the GO-PEG system, the peak density thereof reaches approximately 0.12 g/cm^3^, which is 20% higher than the peak density observed in the pure GO system (approximately 0.10 g/cm^3^). Additionally, the peak in the GO-PEG system is narrower, indicating a more localized and concentrated interaction zone for HOCl, with a density of approximately 0.043 g/cm^3^. This sharper profile suggests enhanced stabilization and stronger confinement of HOCl molecules within the GO-PEG structure.

For the GO-PEI system (Figure 9C), the density is even higher (approximately 0.14 g/cm^3^) compared to GO and GO-PEG, which suggests stronger interactions, likely due to the amine groups in PEI that have an electrostatic interaction with HOCl.

The heatmaps (Figure 9; right) display the corresponding 2D area density of the systems in the X–Z plane, providing a spatial view of where the densest regions of GO and HOCl are located. The central red region corresponds to the highest density.

A comparison between the heatmap of the GO-HOCl (Figure 9A) and GO-PEG-HOCl systems (Figure 9B) reveals distinct differences in the interaction and retention of HOCl. The GO-HOCl system exhibits a broader density profile, indicating a more dispersed interaction of HOCl across the X- and Z-axes. This weaker interaction aligns with the results from the line graph (Figure 9A, left), where the HOCl density was not as sharply peaked near GO. In contrast, in the GO-PEG-HOCl system, the central density (red region) is high and with a slightly sharper and more confined peak, with the density localized to a narrower region (Figure 9B, right). This narrower profile highlights the enhanced retention and stronger interaction of HOCl within the GO-PEG matrix. The results demonstrate that PEG functionalization significantly improves the ability of GO to localize and stabilize HOCl.

For GO-PEI, Figure 9C (right), the central density (red zone) has higher intensity and is more localized compared to GO alone and displays a similar pattern as GO-PEG.

#### 3.6.4 Energy calculations

**FIGURE 10:**
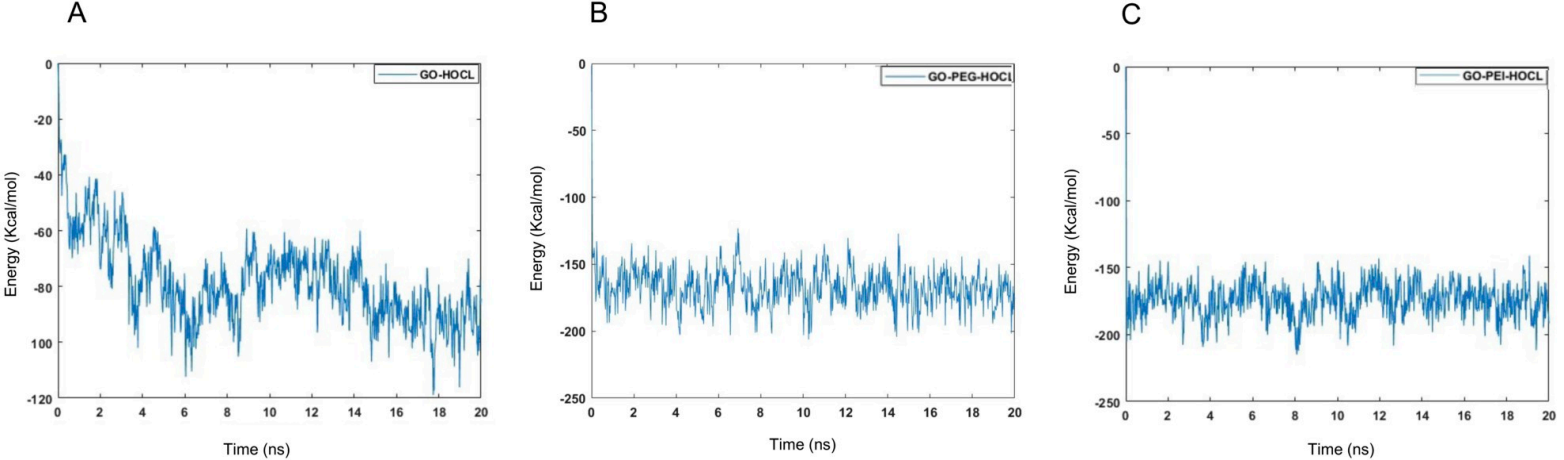
Calculation of the interaction energy between HOCl and GO **(A)**, GO-PEG **(B)**, and GO-PEI **(C)** during 20-ns MD simulations.

The energy plots presented in Figure 10 represent the interaction energy between HOCl and the different systems (GO, GO-PEG, and GO-PEI) over the 20-ns MD simulations.

In Graph A (GO-HOCl), the energy fluctuates significantly between −120 kcal/mol and 0 kcal/mol, indicating less stability and weaker interactions. The system shows large variations, particularly in the first 5 ns, followed by some stabilization after 14 ns. In contrast, Graphs B (GO-PEG-HOCl) and C (GO-PEI-HOCl) display much lower energy ranges, with averages between −173 ± 25 kcal/mol and −179 ± 25 kcal/mol, respectively, signifying stronger interactions and greater stability especially in the GO-PEI system, likely due to strong Coulombic forces (e.g*.,* electrostatic attraction between amine groups in PEI and HOCl). These interactions enhance the adsorption and stabilization of HOCl near the GO surface, which is consistent with the higher density peak and lower interaction energy observed in the simulation.

In contrast, the GO-PEG system lacks charged functional groups but contains flexible polyethylene glycol chains that introduce a highly hydrophilic environment around the GO surface. These PEG chains can promote hydrogen bonding and steric confinement of HOCl molecules within the polymer layer, leading to increased retention of HOCl compared to GO. However, because PEG does not provide strong electrostatic attraction, the interaction strength remains lower than that observed in the GO-PEI system.

The comparative molecular dynamics simulations reveal a distinct behavior of the three systems in terms of molecular interaction, spatial density distribution, and energy stability. The GO-HOCl system exhibited the weakest interaction with HOCl, characterized by broad density profiles, dispersed heatmap patterns, and fluctuating energy values. This indicates less effective retention and weaker adsorption mechanisms. In contrast, the functionalized systems, GO-PEG-HOCl and GO-PEI-HOCl, demonstrated enhanced interaction and stability. The GO-PEG-HOCl system showed a narrower density profile, higher peak density, and more confined heatmap regions, indicating improved retention and localized stabilization of HOCl molecules. Energy plots for this system confirmed the higher stability. Similarly, the GO-PEI-HOCl system exhibited a strong interaction, with the highest density peak and intense and localized heatmap patterns comparable to GO-PEG-HOCl. The presence of amine groups in PEI likely facilitates stronger electrostatic interactions with HOCl, as reflected in the stable energy profile and minimal fluctuations.

## 4 Conclusion

In summary, GO reduced the number of viable cells, yet it was subject to biodegradation by granulocyte-like cells, their NETs, and MPO, a crucial enzyme present in NETs. Despite this biodegradability, cell viability remained lower than in cells treated with GO-PEG and GO-PEI. Granulocyte-like cells contributed to GO degradation through degranulation, NET release, and phagocytosis. Notably, GO-PEG exhibited higher cell viability compared to GO, positioning it as a promising carrier for drug delivery, but Raman mapping did not confirm its biodegradation. GO-PEI showed improved cell viability, indicating better biocompatibility than that of GO, and it was, in addition, degraded by both NETs and MPO. The significant changes in MPO’s secondary structure upon the addition of H_2_O_2_ to MPO/GO and MPO/GO-PEG blends suggest that the degradation products interact with MPO differently than the intact GO and GO-PEG, which do not undergo catalytic degradation in the absence of H_2_O_2_. In contrast, the interaction with GO-PEI caused only minor perturbations in MPO’s structure, suggesting that HOCl, a product of MPO catalysis, plays a crucial role in the effective biodegradation of GO-PEI. Molecular dynamics simulations also conclusively demonstrate that functionalizing GO with PEG and PEI significantly improves its adsorption and retention capabilities for HOCl. These findings underscore the complex mechanistic differences in MPO-mediated biodegradation pathways, influenced by the specific physicochemical properties of the GO-based nanomaterials.

Although the present study provides important insights into the interaction and biodegradation of functionalized GO by myeloperoxidase and granulocyte-like cells, several limitations should be acknowledged. We primarily investigate these processes under *in vitro* conditions, which may not fully replicate the complexity of biological environments *in vivo*. Therefore, further studies involving *in vivo* models and complementary structural characterization techniques will be necessary to better understand the long-term biodegradation behavior and biological responses to functionalized graphene-based materials.

## Data Availability

The datasets presented in this study can be found in online repositories. The names of the repository/repositories and accession number(s) can be found below: Model structures, simulation trajectories, and energetics of the different systems described herein are freely available through Zenodo.org, with DOI: 10.5281/zenodo.14826185.
