# Supplementary material for: Comparative biodegradation of functionalized graphene oxide nanosheets by myeloperoxidase and neutrophil extracellular traps
Source: Front Bioeng Biotechnol. 2026 May 13;14:1797692. doi: 10.3389/fbioe.2026.1797692 (PMC13213391; doi:10.3389/fbioe.2026.1797692)
Supplement: Supplementary file 1 [file Supplementaryfile1.docx]

**Supplemental Fig. 1.** Representative AFM images of GO and its derivatives. Height profile of white lines in images is shown besides each AFM image.

**Supplemental Fig. 2.** HL60 cells differentiation to granulocyte-like cells after 5 days of treatment with 70 mM DMF. A) HL60 cells, B) granulocyte-like cells. The cells stained with May-Grünwald′s eosine-methylene blue solution modified.

**Supplemental Fig. 3.** Bright field microscopy of HL60 cells differentiation to granulocyte-like cells after 5 days of treatment with 70 mM DMF. The normal cells on the top (-DMF) and the cells with multilobulated nuclei shape appeared in below picture (+DMF).

**Supplemental Fig. 4.** Bright field microscopy of granulocyte-like cells exposed to 200 µg/mL concentration GO, GO-PEG, and GO-PEI for 48 h.


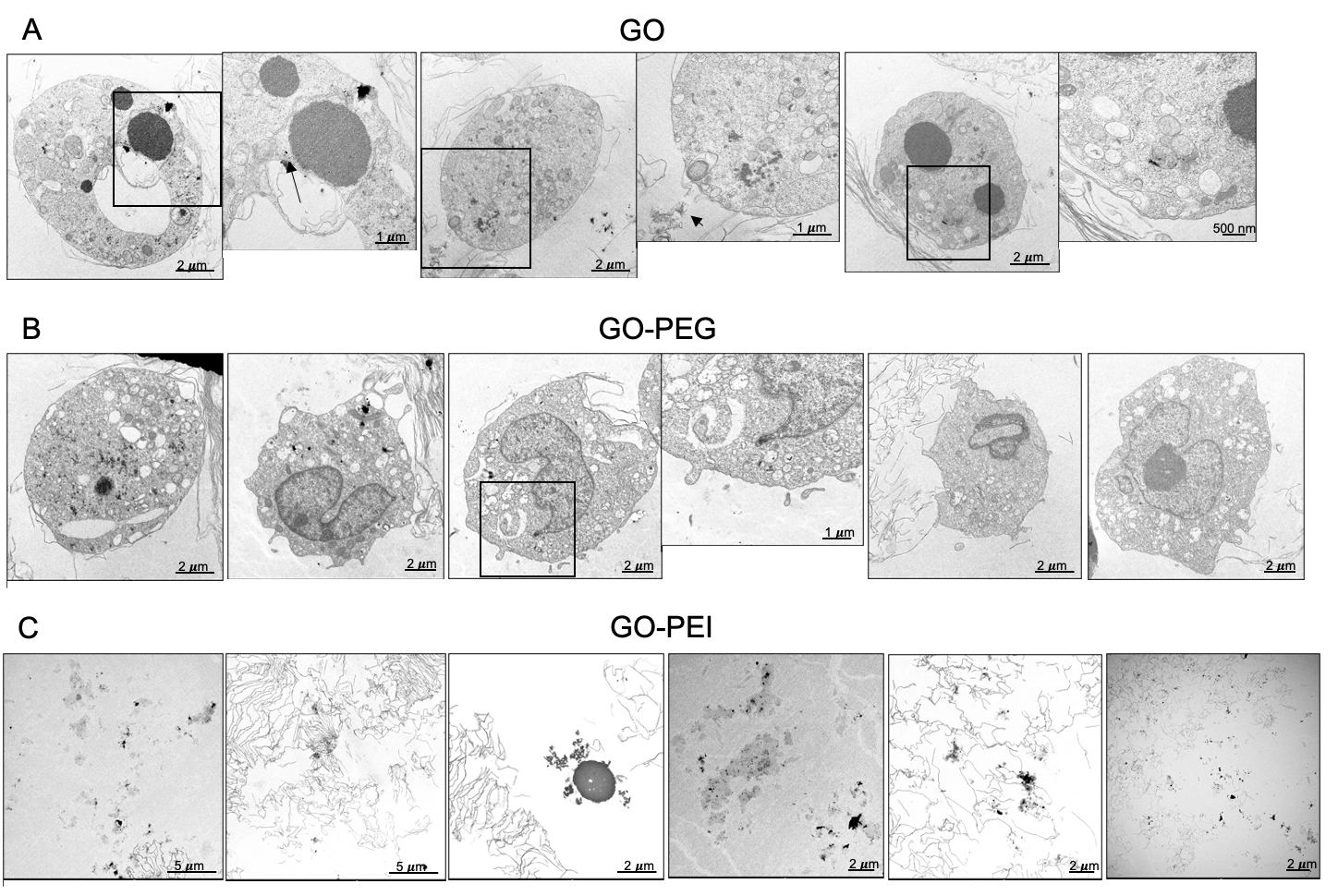


**Supplemental Fig. 5.** Additional TEM images on granulocyte-like cells exposed to 200 µg/mL concentration GO, GO-PEG, and GO-PEI for 24 h.


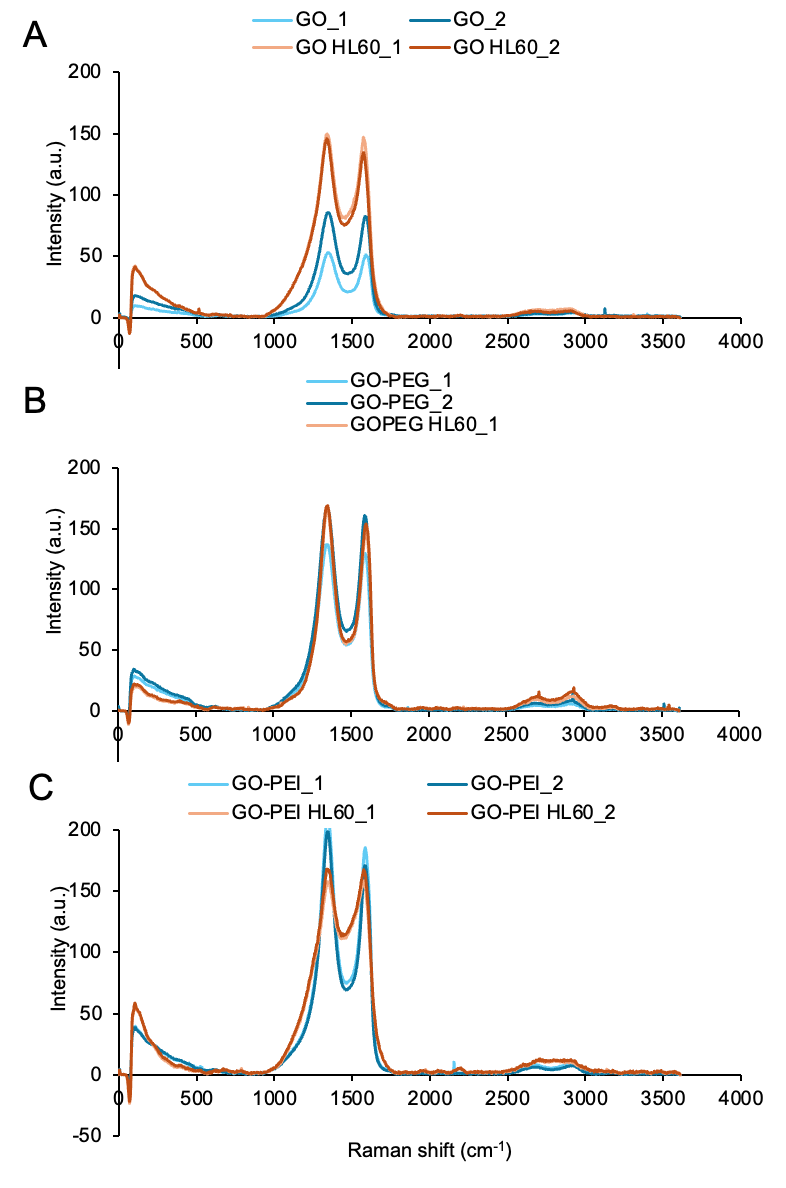


**Supplemental Fig. 6.** Effect of HL60 cells on Raman spectra of A) GO, B) GO-PEG, and C) GO-PEI. HL60 cells exposed to 400 µg/mL concentration of GO, GO-PEG, and GO-PEI. Each graph represents the average Raman spectra of 450 points located in 1 µm distance from each other in a rectangle area of 30 µm ×15 µm on GO, GO-PEG, and GO-PEI samples treated with HL60 cells marked with “HL60” (orange and red curves). Two different regions marked with _1 and _2 was shown. The control samples are GO, GO-PEG, and GO-PEI without any cell and without any incubation in the culture medium (light and dark blue curves).


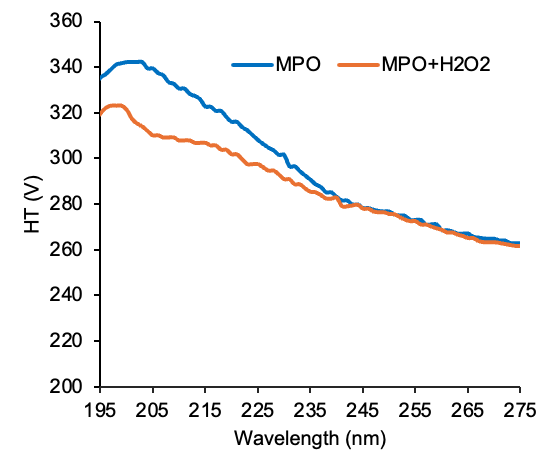


**Supplemental Fig. 7.** High Tension (HT) spectra measurements from CD spectroscopy of MPO, both with and without the addition of H_2_O_2_.


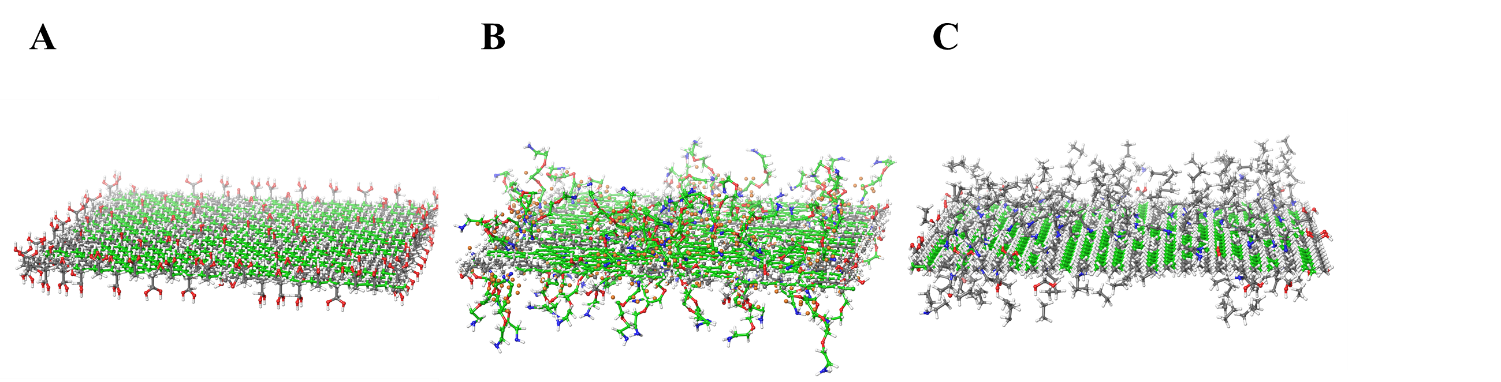


**Supplemental Fig. 8.** Structural representation of GO (A), GO-PEG (B) and GO-PEI (C), illustrations the incorporation of hydroxyl (-OH), carboxylic acid (-COOH), and epoxide (-O) groups in GO, as well as the covalent attachment of PEG and PEI to enhance biocompatibility and functionality.


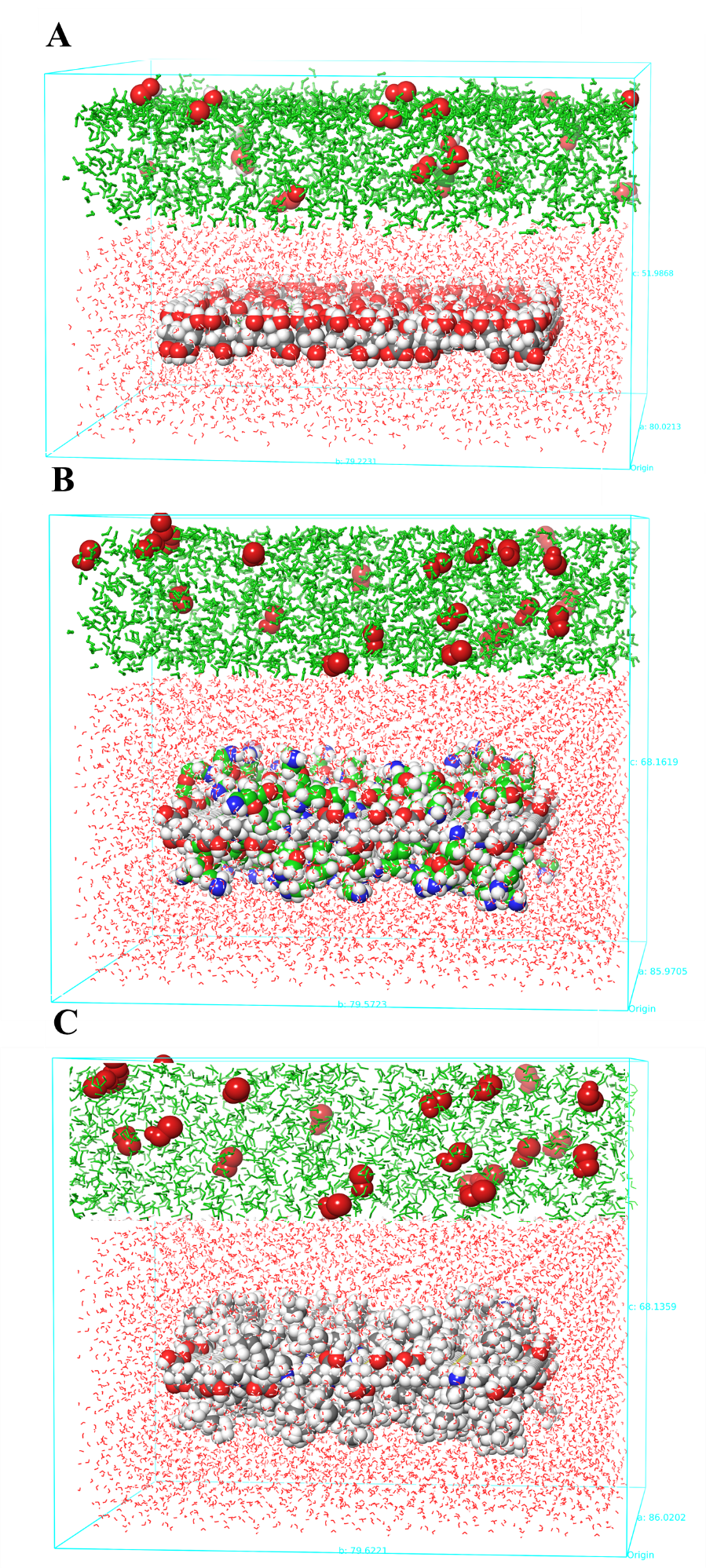


**Supplemental Fig. 9.** Representative initial configurations of the solvated GO - HOCl system (A), and the corresponding GO-PEG (B) and GO-PEI (C) conjugates, displayed in the lower parts of each figure. The GO systems are shown in space-filling models and waters ias red/white stick models. The added mixtures of HOCl molecules (red) solvated in water (green) are shown at the top of each box.

**Supplemental Table 1.** The FTIR peaks and interpretation relevant to Fig. 1A.

| **Peak (cm⁻¹)** | **Interpretation** |
| --- | --- |
| **~3620** | O–H stretching |
| **~3250** | N–H stretching |
| **~2950** | C–H stretching |
| **~1635** | C=C stretching |
| **~1380** | C–N stretching |
| **~1070** | C–O stretching |
| **~1035** | C–O stretching |
